# 

**Published:** 1899-04

**Authors:** 


### Communicated.

### SEVENTH DISTRICT DENTAL SOCIETY OF NEW YORK.

The annual meeting of the Seventh District Dental Society of the State of New York will be held in the assembly room of the New Osborn House, Rochester, New York, Tuesday and Wednesday, April 27th and 28th. Meeting will be called to order at 10 o'clock Tuesday.

PROGRAMME.

1. Presidents annual address, Dr. B. S. Hert, Rochester.

2. What shall we do to increase the attendance of our Society meetings? Dr. F. A. Greene, Geneva. Discussion opened by Dr. W. A. White, Phelps.

3. Practical Hints, Dr. F. W. Proseus, Rochester. Discussion opened by ——————

4. Wherein does a country Dentist differ from a city Dentist? Dr. James Dennison, Waterloo. Discussion opened by Dr. J. A. Burkhart, Dansville.

5. Dental Ethics, Dr. H. S. Miller, Rochester. Discussion opened by Dr. Chas. T. Howard, Rochester.

6. Observations on adenoid growths and thumb sucking in producing irregular dentition, W. W. Skinner, M. D., Rushville. Discussion opened by Sumner Hayward, M. D., Rochester.

7. Character as expressed by the Maxillæ and Teeth, Dr. J. Wright Beach, Buffalo. Discussion opened by Dr. Frank French, Rochester.

8. Preparatory work in artificial Dentures, Dr. J. F. Knapp, Geneva. Discussion opened by J. H. Beebe, Rochester.

9. Aseptics and Aseptisis, Dr. Frank Sibley, Rochester. Discussion opened by Dr. ——————

10. A talk on Cataphoresis, Dr. R. H. Hofheinz, Rochester. Discussion opened by Dr. W. W. Smith, Penn Yan.

11. Weld's Chemico-Metalic method of filling root canals, Dr. F. H. Lee, Auburn. Discussion opened by Mr. D. H. Piffard, Piffard.

12. A talk on the "X Ray" and a practical demonstration of their application in oral and general surgery, by Mr. John Dennis, Rochester.

Mr. Dennis will present some entirely new and original applications of the X-Rays in surgery. The apparatus will be the latest and best.

It is expected that this the demonstration of the X-Ray will be the most complete *ever presented.*

There will be a display of dental goods and appliances.

The New Osborn House will be the official headquarters. A rate of $2.00 per day has been made by the management. First come, first served will be the rule, but rooms may be reserved on or after April 15th, by addressing New Osborn House, Rochester, N. Y.

W. W. BELCHER,
Chairman, Business Committee.

---

### Editorial, Etc.

### To Readers and Correspondents.

Communications intended for publication, and exchange journals and papers, should be addressed to the Editor of THE AMERICAN JOURNAL OF DENTAL SCIENCE, No. 845 N. Eutaw St. Baltimore, Md. All letters relating to the business of the JOURNAL, or containing cash remittances, to be directed to Snowden & Cowman Mfg. Co., 9 W. Fayette Street, Baltimore, Md. Articles for publication should be received by the Editor at least two weeks previous to the first of the month on which the number in which it is designed for them to appear is issued.

☞ THE AMERICAN JOURNAL OF DENTAL SCIENCE will contain fifty pages of reading matter in each number, making a volume of about Six Hundred pages,

EXCLUSIVE OF ADVERTISEMENTS.

